# Reactive Postural Responses After Mild Traumatic Brain Injury and Their Association With Musculoskeletal Injury Risk in Collegiate Athletes: A Study Protocol

**DOI:** 10.3389/fspor.2020.574848

**Published:** 2020-10-29

**Authors:** Amanda Morris, Benjamin Cassidy, Ryan Pelo, Nora F. Fino, Angela P. Presson, Daniel M. Cushman, Nicholas E. Monson, Leland E. Dibble, Peter C. Fino

**Affiliations:** ^1^Department of Health and Kinesiology, University of Utah, Salt Lake City, UT, United States; ^2^Department of Physical Therapy and Athletic Training, University of Utah, Salt Lake City, UT, United States; ^3^Division of Epidemiology, Department of Internal Medicine, University of Utah, Salt Lake City, UT, United States; ^4^Division of Physical Medicine and Rehabilitation, University of Utah School of Medicine, Salt Lake City, UT, United States; ^5^Department of Orthopaedic Surgery Operations, University of Utah School of Medicine, Salt Lake City, UT, United States

**Keywords:** wearable sensors, push and release test, return-to-play, compensatory stepping, concussion

## Abstract

**Background:** Deficits in neuromuscular control are widely reported after mild traumatic brain injury (mTBI). These deficits are speculated to contribute to the increased rate of musculoskeletal injuries after mTBI. However, a concrete mechanistic connection between post-mTBI deficits and musculoskeletal injuries has yet to be established. While impairments in some domains of balance control have been linked to musculoskeletal injuries, reactive balance control has received little attention in the mTBI literature, despite the inherent demand of balance recovery in athletics. Our central hypothesis is that the high rate of musculoskeletal injuries after mTBI is in part due to impaired reactive balance control necessary for balance recovery. The purpose of this study is to (1) characterize reactive postural responses to recover balance in athletes with recent mTBI compared to healthy control subjects, (2) determine the extent to which reactive postural responses remain impaired in athletes with recent mTBI who have been cleared to return to play, and (3) determine the relationship between reactive postural responses and acute lower extremity musculoskeletal injuries in a general sample of healthy collegiate athletes.

**Methods:** This two-phase study will take place at the University of Utah in coordination with the University of Utah Athletics Department. Phase 1 will evaluate student-athletes who have sustained mTBI and teammate-matched controls who meet all the inclusion criteria. The participants will be assessed at multiple time points along the return-to-play progress of the athlete with mTBI. The primary outcome will be measures of reactive postural response derived from wearable sensors during the Push and Release (P&R) test. In phase 2, student-athletes will undergo a baseline assessment of postural responses. Acute lower extremity musculoskeletal injuries for each participant will be prospectively tracked for 1 year from the date of first team activity. The primary outcomes will be the measures of reactive postural responses and the time from first team activity to lower extremity injury.

**Discussion:** Results from this study will further our understanding of changes in balance control, across all domains, after mTBI and identify the extent to which postural responses can be used to assess injury risk in collegiate athletes.

## Introduction

Standing balance problems are well documented in collegiate athletes after mild traumatic brain injury (mTBI) (Guskiewicz, 2011; Broglio et al., 2014). However, the Balance Error Scoring System (BESS), the most common clinical balance assessment for a suspected mTBI, only subjectively measures postural control in a variety of static conditions (Riemann and Guskiewicz, 2000). The BESS and other clinical tests, such as the standard Romberg test (Guskiewicz, 2011), struggle to detect subtle balance deficits that persist throughout an athlete's recovery from mTBI (Buckley et al., 2018) and therefore may be limited in their ability to determine return-to-play (RTP) readiness. Additionally, postural demands during athletic pursuits are not static. Recent studies suggest clinical testing of dynamic balance using the timed tandem gait test has clinical utility in mTBI management (Oldham et al., 2018). However, other aspects of balance control remain untested in mTBI management.

Balance control can be separated into three domains based on the goal of the task: (1) maintaining a posture (e.g., standing balance), (2) transitioning between postures with voluntary movement (e.g., gait, turning, gait initiation/termination, standing from a chair), or (3) restoring stability (e.g., recovering from a trip, slip, or push) (Pollock et al., 2000). Objective measures of standing balance from instrumented force platforms (DeBeaumont et al., 2011; Quatman-Yates et al., 2015; Fino et al., 2016b) and inertial sensors (King et al., 2014; Parrington et al., 2019) suggest maintenance of posture can remain impaired beyond the clinical RTP decision in athletes, and gait and turning abnormalities suggest postural control during transitions may also be affected for up to 1 year after mTBI (Fino et al., 2016a, 2018). Yet, there is limited knowledge of how mTBI affects the restoration of stability after loss of balance. There is evidence to suggest that mTBI history affects stability after dynamic movement in athletes (Lynall et al., 2020). However, no study has examined reactive stabilization following an unexpected external disturbance in athletes after mTBI. Considering athletes are frequently required to recover balance after an external perturbation or sudden destabilizing event, examining the effects of mTBI on restoring stability is important for a comprehensive understanding of the impact of mTBI in real-world and sporting environments.

Reactive postural responses rely on short-, medium-, and long-latency responses, resulting from interaction among spinal circuits, the brainstem, and the cerebral cortex. The short-latency response is likely spinally mediated and is too small to stabilize balance, whereas medium- and long-latency responses are mediated by the brainstem and cortex and stabilize balance via whole-body, synergistic muscle activations (Jacobs and Horak, 2007). During change in support responses in particular, a transcortical loop through the motor cortex is involved in initiation (Jacobs and Horak, 2007). Yet, the response to a perturbation is also dependent on the preperturbation central set, which is the neuromotor state resulting from changes in initial contexts such as changes in cognitive state or sensorimotor conditions (Jacobs and Horak, 2007). Prior to a loss of balance, cortical loops update the central set to prime postural responses (Jacobs and Horak, 2007; Mochizuki et al., 2008) in an anticipatory manner according to stored visual information about the surrounding environment (Maki et al., 2001; Zettel et al., 2005, 2008a,b). Because attentional resources may be required to maintain an appropriate central set, concurrent cognitive demands can limit this anticipatory preparation; attentional resources switch from the concurrent cognitive task to the balance-recovery task after the postural disturbance (Maki and McIlroy, 2007), and delayed set-switching can compromise the ensuing stepping response (Maki et al., 2001). Concurrent cognitive tasks also attenuate the N1 perturbation-evoked response within the medial prefrontal cortex (Quant et al., 2004) associated with task-switching (Rushworth et al., 2002). Persistent executive and attentional dysfunction (Howell et al., 2013b) and lasting structural and functional neuroimaging abnormalities within the frontal and prefrontal cortex (Chamard et al., 2016; Churchill et al., 2017a,b,c; Bigler, 2018), may limit the cognitive reserve (Stern, 2009) for completing simultaneous tasks after mTBI and could be responsible for impaired dual-task balance. Based on these previous findings, we posit that persistently slower set-switching reaction times and larger switch costs after mTBI (Mayr et al., 2014; Moore et al., 2014) may adversely affect balance recovery, particularly in highly dynamic sporting environments with changing motor and cognitive demands. While dual-task paradigms involving simultaneous static or dynamic balance and cognitive tasks elicit large balance deficits in individuals with mTBI (Howell et al., 2013a; Lee et al., 2013; Register-Mihalik et al., 2013; Fino et al., 2018), the influence of cognitive demands on the ability to restore stability remains unclear after mTBI.

Numerous research groups suspect that there is a link between impaired neuromuscular control and musculoskeletal injuries after mTBI. Notably, both of these aspects have solid grounding in the literature; deficits in neuromuscular control after mTBI are widely reported in the literature (Howell et al., 2018b; Wilkerson et al., 2018) and musculoskeletal injuries are around two times more likely after mTBI (Nordström et al., 2014; Lynall et al., 2015, 2017; Pietrosimone et al., 2015; Brooks et al., 2016; Cross et al., 2016; Gilbert et al., 2016; Herman et al., 2017; Fino et al., 2019; Reneker et al., 2019). Recently, dual-task gait impairment after mTBI has been linked to musculoskeletal injury risk (Howell et al., 2018a). Despite the overlap and apparent connection, a concrete connection between post-mTBI deficits and musculoskeletal injuries has yet to be established. Although absent from mTBI literature to date, imprecise responses following a loss of balance may contribute to the high rate of musculoskeletal injuries after mTBI. In healthy collegiate athletes, postural responses following the release of a sustained force at the trunk, with the lower body fixed, are highly associated with future injuries to the low back (Cholewicki et al., 2005) and knee (Zazulak et al., 2007). Disrupted perception–action coupling has been suggested as an alternative potential mechanism for post-mTBI musculoskeletal injury. This theory suggests that neurophysiological dysfunction stemming from mTBI dysregulates the perception–action coupling loop (Eagle et al., 2020); misestimating one's abilities (both the participant's physical abilities related to the environment and the participant's perception based on repeated action in the environment) may manifest in improper timing of movement or body position and increase behavioral risk after mTBI (Eagle et al., 2020). However, direct experimental evidence is lacking.

Two major challenges in establishing the connection between musculoskeletal injury risk and neuromuscular deficits are the lack of sensitivity of assessments and obtaining adequate sample size. Clinical tests often lack sensitivity and specificity, particularly to subtle motor deficits that may have practical implications. There is growing interest in using neuroimaging and serum biomarkers to examine changes in cerebrovascular reactivity (Churchill et al., 2020) and systemic inflammation (Di Battista et al., 2019) after mTBI, but a direct connection between mTBI and musculoskeletal injury risk remains unclear. In a recent study, several clinical tests including the standard assessment of concussion (SAC), BESS, Immediate Post-Concussion Assessment and Cognitive Training (ImPACT), clinical reaction time, and the King-Devick failed to predict musculoskeletal injury risk after mTBI (Buckley et al., 2020). As a consequence, more sophisticated tests and instrumentation are often used to determine post-mTBI neuromuscular deficits. These assessments, often conducted within biomechanical laboratories, are not feasible in clinical settings, a necessary requirement for adequate power and future clinical translation. Recent technological advances in wearable, inertial sensors may be capable of addressing these limitations by enabling objective and sensitive assessments in clinical settings (Horak et al., 2015; Nouredanesh and Tung, 2015; El-Gohary et al., 2017). For example, instrumenting the BESS with a single inertial sensor identified acute mTBI more accurately than the clinical error count (King et al., 2017; Parrington et al., 2018). Inertial sensors can identify subtle deficits in gait, distinguishing between healthy controls and individuals within 72 h of mTBI and up to 2 weeks postinjury (Howell et al., 2015). Recently, objective qualities of gait, quantified with inertial sensors, differed between athletes with mTBI who went on to sustain a future lower extremity musculoskeletal injury and those who did not (Oldham et al., 2020). Similarly, worsening dual task cost of walking speed throughout mTBI recovery was associated with risk of incurring a sport-related injury during the year following mTBI (Howell et al., 2018a). Because inertial sensors are portable and clinically feasible, they can be used to facilitate clinical testing on large numbers of collegiate athletes.

Similar to static balance and gait, clinical assessments of reactive postural responses in other populations rely on scoring guidelines based on visual observation, but wireless, wearable inertial sensors can improve the accuracy of these assessments. For example, inertial sensors can detect differences in reactive postural responses between disease states [healthy control, multiple sclerosis (Smith et al., 2016; El-Gohary et al., 2017), Parkinson disease (Smith et al., 2016)]. Wearable sensors also provide information about quality of movement using a variety of objective characteristics. However, no study has used inertial sensors to quantify reactive postural responses in clinical mTBI settings. Improved understanding of how mTBI impacts the quality of balance recovery is critical to determine the implications of persistent balance impairments after mTBI, such as increased injury risk, and to develop clinical rehabilitation strategies to optimize athletes' neuromuscular control prior to return to competitive activity.

Our central hypothesis is that the high rate of musculoskeletal injuries after mTBI is in part due to impaired postural responses and balance recovery. Therefore, the purpose of this study is to (1) characterize reactive postural responses in athletes with recent mTBI compared to healthy control subjects, (2) determine the extent to which postural responses remain impaired in athletes with recent mTBI who have been cleared to RTP, and (3) determine the relationship between postural responses and acute lower extremity musculoskeletal injuries in a general sample of healthy collegiate athletes. We hypothesize that (1a) athletes with recent mTBI will take longer to regain balance during assessments of reactive postural responses compared to control athletes; (1b) athletes with recent mTBI will have larger deficits, in postural responses when simultaneously performing a dual task, relative to single task; (2) athletes with recent mTBI will improve reactive postural responses but will still demonstrate deficits compared to control subjects at all time points; (3a) that impaired postural responses at a baseline assessment will be associated with a faster time to acute musculoskeletal injury in healthy collegiate athletes; and (3b) objective measures of reactive postural responses will be better predictors of musculoskeletal injuries compared to clinical measures.

## Methods

This study has two phases that will run concurrently. Phase 1 includes the first two aims: (1) to evaluate acute differences in postural responses after mTBI and (2) to evaluate persistent mTBI-related deficits in postural responses after RTP. Phase 2 contains the third aim: to characterize associations between baseline variations in postural responses and prospective lower extremity injuries in healthy athletes.

### Phase 1: Characterization of Postural Responses After mTBI and Persistent mTBI-Related Deficits

#### Participants

For phase 1, 80 subjects will be recruited (40 athletes who experienced recent mTBI and 40 healthy teammate controls, matched on position and practice exposure). Sports medicine fellowship–trained team physicians with at least 10 years of training and clinical experience will diagnose mTBI based on history and physical examination, which is currently the gold standard. All subjects will be between the ages of 18 and 30 years and tested on site in the Athletic Training Clinics at the University of Utah. Informed written consent will be obtained from all participants.

#### Inclusion and Exclusion Criteria

For phase 1, inclusion criteria for mTBI recruitment are (1) age 18 to 30 years, (2) current participant in NCAA Division I athletics, and (3) mTBI within last 10 days (subjects must be assessed before beginning RTP protocol, even if acute time point is missed). Exclusion criteria for mTBI subjects are (1) previous history of vestibular or somatosensory pathology, (2) history of major injury to either leg requiring surgery within the last 2 years, and (3) body mass index (BMI) >40 kg/m^2^. Inclusion criteria for matched controls are (1) age 18–30 years, (2) current participant in NCAA Division I athletics. Exclusion criteria for healthy control subjects are (1) previous history of vestibular or somatosensory pathology, (2) recent mTBI (<1 year), (3) history of major injury to either leg requiring surgery within the last 2 years, and (4) BMI >40 kg/m^2^.

#### Assessment Procedures

For phase 1, athletes will be assessed at four time points: (1) within 48 h of mTBI (acute), (2) within 24 h of starting RTP protocol (PreRTP), (3) within 24 h of being medically cleared for RTP (PostRTP), and (4) ~6 months after injury (6Month). Assessments will take ~15 min and will be administered at the end of current Pac-12 CARE Affiliated Project (CAP) testing (Broglio et al., 2017). CAP testing is a tablet-based battery of measures that examine personal and family health history, neurocognitive assessments, neurological status, postural stability, symptoms, and oculomotor/oculovestibular function. Tests included in CAP testing are the Sport Concussion Assessment Tool, Fifth Edition; BESS; Brief Symptom Inventory 18; SAC; ImPACT; and EYESYNC (SyncThink, Palo Alto, CA) measures (Maruta et al., 2018). Assessments of matched control subjects will be tested within 24 h of the testing session of the athlete with mTBI. Return to play will follow guidelines set forth by the University of Utah Concussion Management Plan. Athletes will progress to RTP when, under the guidance of the physician and athletic trainer, the assessment battery including symptom assessment, ImPACT, and BESS has normalized. In the current study, athletes will be cleared to start RTP by only a limited number of team physicians.

Within 48 h from the time of the mTBI, informed written consent will be obtained from the athlete with mTBI and the matched control teammate. A total of 40 athletes with mTBI and 40 matched control athletes will complete assessments at four time points (Acute, PreRTP, PostRTP, 6Month). Each assessment will include questionnaires, clinical tests of function, and instrumented assessments of reactive postural responses (Table 1).

**Table 1:** Questionnaires, surveys, and tests utilized in the present study with description of subject group (mTBI, control, both) and time point (Acute, PreRTP, PostRTP, 6Month, all).

**Validated Questionnaires**	**Purpose**	**Subject group**	**Time point**
Multidimensional fatigue inventory	Multiple domains of fatigue	Both	All
Pittsburgh sleep quality index	Sleep quality and disturbances and daytime dysfunction	Both	All
Injury-psychological readiness to return to sport scale	Confidence to return to play	mTBI	All
Return to sport after serious injury questionnaire	Motivation underlying return to play	mTBI	6 month
**Patient self-report**	**Purpose**	**Subject group**	**Time point**
Questions adapted from the OSU TBI-ID	Concussion history	Both	Acute, 6 month
Questions regarding recent (<2 years) lower extremity musculoskeletal injury and surgery history	Lower extremity injury and surgery history	Both	All
Questions regarding return-to-learn progress	Return-to-learn	mTBI	All
Questions regarding medication use postconcussion	Medication use	mTBI	All
**Test**	**Purpose**	**Subject group**	**Time point**
Clinical reaction time (drop stick test)	Clinical reaction time	Both	All
Instrumented balance error scoring system	Static balance	Both	All
Push and release test	Reactive postural response	Both	All

##### Questionnaires

At every time point, the athlete with mTBI will complete the following questionnaires: Multidimensional Fatigue Inventory (MFI), Pittsburgh Sleep Quality Index (PSQI), Injury-Psychological Readiness to Return to Sport (I-PRRS) scale. The MFI is a 20-item questionnaire designed to measure the following domains of fatigue: general fatigue, physical fatigue, mental fatigue, motivation, and activity (Smets et al., 1995). The PSQI is a 19-item questionnaire designed to assess sleep quality, sleep disturbances, and daytime dysfunction. In the present study we will exclude the five questions rated by the bed-partner or roommate (Buysse et al., 1989). The I-PRRS scale is a sport psychometric test to assess injured athletes' confidence and psychological readiness to return to play (Glazer, 2009). Athletes are asked to rate their confidence on a scale of 0 to 100 on six items related to competing in their sport. To determine motivation underlying return to sport, at the 6Month time point, the athlete with mTBI will also complete the 21-item Return to Sport after Serious Injury Questionnaire (Podlog and Eklund, 2005). Information regarding return-to-learn experience and medication use after mTBI will also be obtained. The matched control athlete will complete the MFI and PSQI at every time point.

##### Clinical reaction time

At every time point, both athletes with mTBI and matched control athletes will perform the Clinical Reaction Time test (RT_clin_). RT_clin_ is a clinical test measuring simple reaction time using an 80-cm dowel with one end inserted in the middle of a hockey puck (Eckner et al., 2010). The participants will sit with their dominant arm on a table, leaving their wrist and hand off the side of the table. The investigator then brings the hockey puck to the level of the athlete's first finger and thumb. After a random duration between 2 and 5 s, the investigator releases the dowel, and the participant attempts to catch the dowel as soon as possible. The distance from the superior aspect of the hockey puck to the superior aspect of the participant's hand is measured. For the present study, two practice trials followed by eight measured trials will be completed by athletes at each assessment.

##### Instrumented BESS

Quiet standing balance will be assessed at every time point using the BESS (Bell et al., 2011). Both athletes with mTBI and matched control athletes will complete this test wearing five inertial measurement units (APDM Inc., Portland, OR) placed on the sternum, lumbar spine, right shank, and right and left feet. The footwear of the athletes will be recorded, and tests will be video recorded. The full BESS battery involves a double-leg stance, single-leg stance (on non-dominant foot), and tandem stance (nondominant behind dominant) performed on both firm and foam surfaces. Each trial will last 30 s, but errors will only be counted during the first 20 s.

##### Push and release reactive postural response paradigm

**Figure 1:**
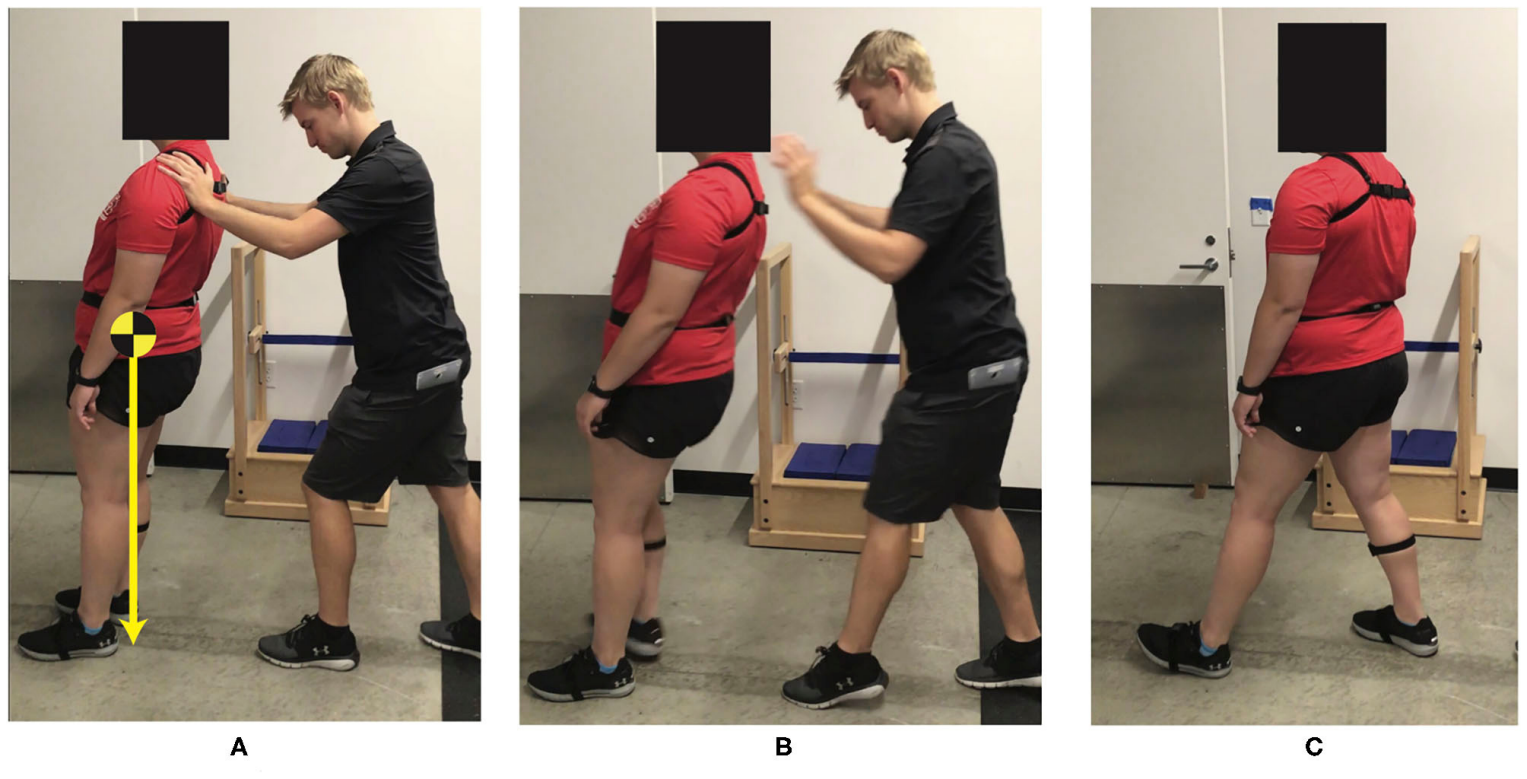
Push and release test **(A)** Prior to release, the subject's center of mass is beyond the subject's base of support (heels). **(B)** Immediately after release of support. **(C)** Subject recovered using one backward step.

Reactive postural responses will be assessed using the Push and Release (P&R) test (Smith et al., 2016; El-Gohary et al., 2017). The P&R is a clinical test that examines reactive postural responses and compensatory stepping behavior after a loss of balance (Jacobs et al., 2006). Prior to each trial, a footplate (8″ long, 5.75″ wide at the toes, 4″ wide at the heels) will be placed on the floor, and the subject will be asked to press their feet firmly and symmetrically against each side of the footplate to ensure a standardized stance width at the beginning of each test. Subjects will lean, while supported by an investigator, until their center of mass is just beyond their base of support (Figure 1, left). After maintaining the supported leaning posture, the investigator will unexpectedly release their hands, requiring the subject to take a step (Figure 1, right). Subjects will perform the P&R, with eyes closed, in four directions: forward, backward, left, and right. Athletes demonstrate faster auditory and visual reaction times than healthy controls (Kaur et al., 2006) and typically perform better on balance tests than nonathletes (Davlin, 2004). Therefore, to increase difficulty of the P&R task for an athletic population, participants will be asked to close their eyes. During competition, athletes are often required to produce appropriate cognitive and motor responses simultaneously. Dual-task assessments are more sensitive to cognitive and motor impairments after mTBI than single-tasks beyond the acute phase of recovery (Register-Mihalik et al., 2013). Therefore, to make the P&R more functionally relevant and sensitive to cognitive and motor impairments, participants will be tested under single- and dual-task conditions. The dual task will be randomized and consists of serial subtraction by 3's, phonemic verbal fluency (FAS test), categorical verbal fluency (animal or fruit naming), and reciting every other letter of the alphabet (Tombaugh et al., 1999). During the dual-task condition, as soon as participants are in the supported leaning posture, they will be prompted with one of the four cognitive tasks. Participants will respond at least three times before being released unexpectedly by the investigator. Inertial measurement units (APDM Inc.) will be placed on the sternum, lumbar spine, right shank, and right and left feet. One sensor will be placed on the investigator's hand to determine release of support time. Data will be collected at 128 Hz and processed using custom-built algorithms in MATLAB (MathWorks, Natick, MA).

#### Outcome Measures

**Figure 2:**
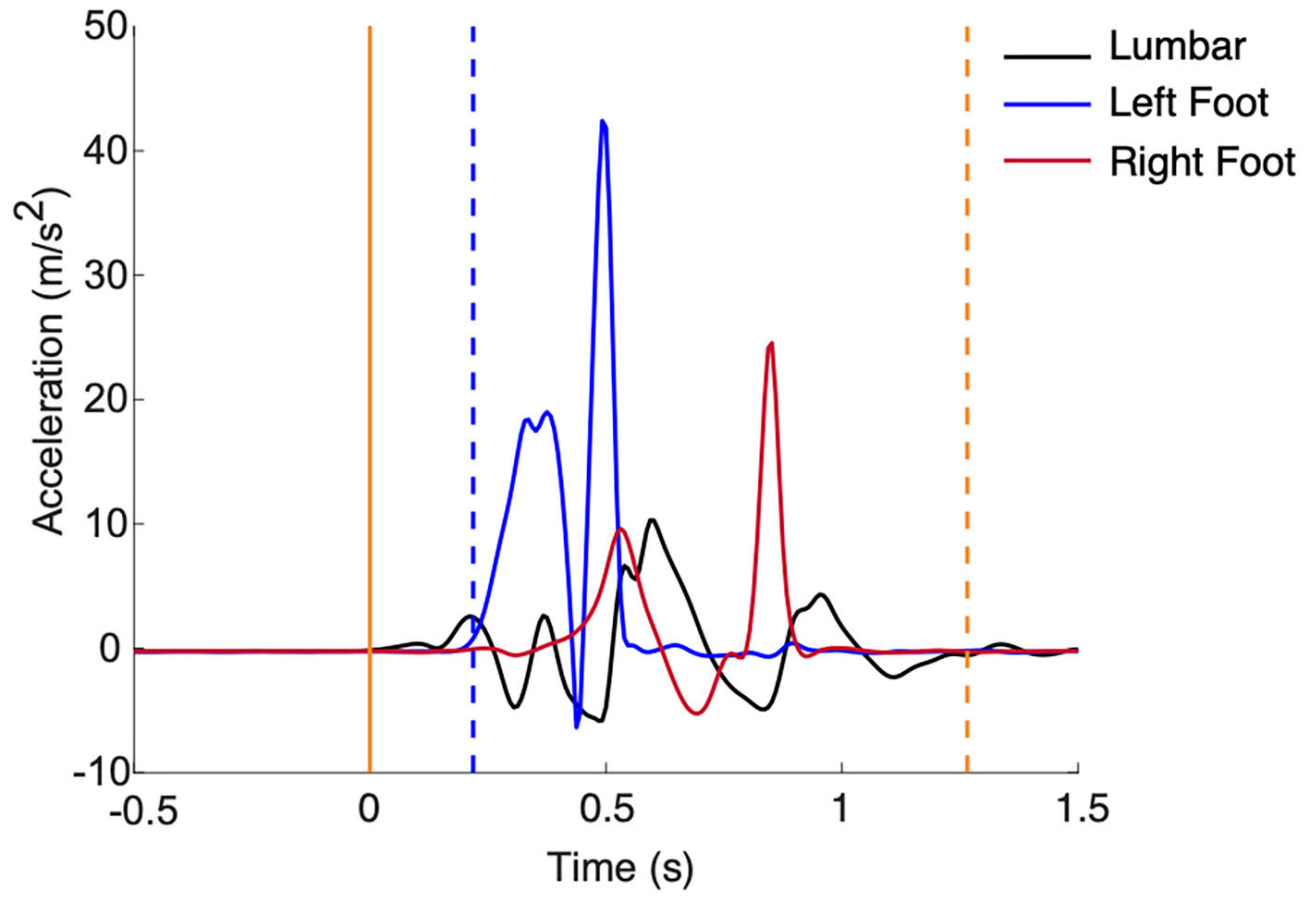
(Left) Support was released at time 0 (solid yellow). Step latency (blue dashed line) and time to stabilization (yellow dashed line) are marked.

Raw acceleration and angular velocity data from the five inertial measurement units will be used to calculate the following outcomes from the P&R test (Figure 2): step latency, step length, and time to stabilization.

Using data from the sensor on the investigator's hand, release of support (*t*_0_) will be identified when acceleration is >5% of gravity. Step latency will be calculated as the amount of time (seconds) from *t*_0_ to first foot movement. First foot movement will be detected when foot acceleration >7% of gravity and rotational rate is >7 degrees/s (El-Gohary et al., 2017). To determine step length (meters) of the first step, the double integral of acceleration of the foot from first foot movement to the next zero velocity instant will be calculated. Magnitude thresholds of 97.4 degrees/s for rotational rate and 0.8 m/s^2^ relative to gravity for acceleration will be used to determine the zero velocity instant (Rebula et al., 2013). Time to stabilization will be calculated as the amount of time (seconds) from *t*_0_ to stabilization. Stabilization will be detected when lumbar acceleration <7% of gravity and rotational rate <7 degrees/s, after the last step (El-Gohary et al., 2017).

The primary outcome measure will be time to stabilization. Secondary outcomes include step latency, step length, and the clinical score (number of steps) (Horak et al., 2009). The clinical score will be rated as follows: 0 = fall, 1 = recovers independently but takes more than 1 step, 2 = recovers independently with one step.

#### Sample Size

The sample size for aim 1 of phase 1 was calculated from differences between symptomatic concussed subjects and healthy controls in response to a tethered pull perturbation (Pan et al., 2015), with an estimated difference of 1.7 standard deviations (Cohen *d* = 1.7) between concussed subjects with residual balance complaints and healthy controls. With an effect size of 1.7, we will have >99% power to detect group differences at acute assessments with a 0.05 significance level and a two-sided *t* test with 40 subjects per group.

The sample size for aim 2 of phase 1 was based on an estimated effect size of Cohen *d* = 0.72 between asymptomatic, previously concussed individuals and healthy controls (Pan et al., 2015). If we observe a similar effect size of 0.72 for the P&R test, we expect 74% power to detect group differences at the primary PostRTP time point using a two-sided *t* test at a Bonferroni-corrected significance level of 0.0125 (0.05/4 for four outcomes: time to stabilization, step latency, step length and clinical score) with 40 subjects per group. Comparisons at PreRTP and 6Month assessments will also be performed but will be considered secondary. We also expect 88% power to detect group differences in the change between acute and subsequent assessments with 40 subjects per group.

#### Statistical Analysis

Aim 1 of phase 1: *t* tests will be used to determine if time to stabilization during the P&R differs between healthy subjects and subjects <48 h after concussion. Linear regression models will assess group differences while controlling for potential confounders of age, sex, contact/noncontact sport, and BMI. Concussion history will be recorded and explored as a covariate if necessary. As clinical scores are ordered with three levels, Wilcoxon Mann-Whitney *U* tests and generalized linear models for ordered multinomial outcomes (ordinal logistic regression) will compare clinical scores between groups. If the clinical scores exhibit a high ceiling effect, scores will be dichotomized and analyzed using χ^2^ tests and logistic models. *t* tests will assess whether concussed athletes had greater differences between single- and dual-task conditions relative to controls.

Aim 2 of phase 1: Bonferroni-adjusted *t* tests will be used to compare groups PostRTP to determine whether athletes with a recent mTBI take longer to stabilize during reactive postural responses after being medically cleared to RTP. Analogous secondary tests will be used for the PreRTP and 6Month assessments. We will also compute the difference in time to stabilization between assessments (e.g., change from Acute to PostRTP) to determine whether concussed athletes improved their postural responses over time. *t* tests will compare the group differences in the change between assessments. Longitudinal analysis using generalized estimating equations (GEEs) with compound symmetry working covariance models will be used to contrast each outcome of the P&R test at each assessment while controlling for lean direction; single vs. dual task; and possible confounders of age, sex, contact/noncontact sport, and BMI. A group-by-assessment interaction will determine if adjusted group differences changed between assessments. Two- and three-way interactions with task (group × task, group × task × assessment) will be included to test for group-difference in task, and if significant, final models will include these interactions. Ordinal logistic regression in a GEE framework will be used to assess changes in clinical scores between assessments.

### Phase 2: Examining the Relationship Between Postural Responses and Acute Lower Extremity Musculoskeletal Injuries

#### Participants

For phase 2, a minimum of 200 subjects will be recruited before the start of their competitive season and prospectively tracked for 1 year. All subjects will be between the ages of 18 and 30 years and tested on site in the Athletic Training Clinics at the University of Utah. Informed written consent will be obtained from all participants.

#### Inclusion and Exclusion Criteria

For phase 2, inclusion criteria are as follows: (1) age 18 to 30 years and (2) current participant in NCAA Division I athletics. Exclusion criteria are as follows: (1) recent (within 6 months) or planned surgery that would result in future time loss and practice/competition exposure and (2) chronic conditions that could confound testing procedures (overuse injuries, medical conditions).

#### Assessment Procedures

Phase 2 assessments will be included as part of preseason physical baseline testing. Acute lower extremity musculoskeletal injuries for each athlete in phase 2 will be prospectively tracked for 1 year from the date of first team activity.

The phase 2 assessment will be identical to the phase 1 matched control athlete assessment. Additionally, mTBI history will be recorded using the Ohio State Traumatic Brain Injury Identification Method (OSU TBI-ID). While the OSU TBI-ID is not made specifically for mTBI, it is well validated (Corrigan and Bogner, 2007; Bogner and Corrigan, 2009) and recommended by the National Institute of Neurological Disorders and Stroke as a Common Data Element for mTBI studies. Lower extremity injury history, including the date of injury, location, type of injury (e.g., sprain, fracture), cause, time lost from sport, and relation of each injury to the athlete's primary sport, will be recorded.

#### Prospective Musculoskeletal Injury Risk

Acute lower extremity musculoskeletal injuries for each athlete in phase 2 will be prospectively tracked for 1 year from the date of their first team activity. Injury data will be queried from the athletes' electronic medical records documented by the University of Utah Athletic Training Staff and maintained for the Pac-12 Sports Injury Registry Management and Analytics Program. Injuries of interest will include any acute orthopedic injury of the lower extremity, pelvis, lumbar spine, or abdomen that resulted in time lost, including but not limited to joint sprains, musculotendinous strains, and fractures. Because abdominal and lumbar muscles are required for stability and movement control (Cholewicki et al., 1997), and the lumbar spine is a critical junction of postural control during sport with high musculoskeletal loads and compressive muscle forces, both lumbar spine and abdominal injuries will be included. Any injury that results in an athlete being unable to fully participate in training or match play will be considered a time loss injury (Fuller et al., 2007; Howell et al., 2018a). Overuse injuries and preexisting conditions will be excluded. The primary outcome measure will be time to first musculoskeletal injury. The number of injuries within 1 year and severity of injuries (time lost from a given injury) will be included as secondary outcomes.

#### Statistical Analysis

Time from first team activity to first musculoskeletal injury will be summarized using Kaplan–Meier methods. Cox proportional hazards models will be constructed to determine if time to stabilization during postural responses is associated with faster times to injury in the general collegiate athlete population (hypothesis 3a). Similar models will be performed for each secondary outcome. Models will first be unadjusted and then potentially adjusted for age, gender, sport, history of a recent musculoskeletal injury, and history of mTBI before enrollment. The number of variables that we will adjust for will depend on the number of musculoskeletal injuries observed. The inclusion of sport as a covariate will account for variation in the biomechanical demands, exposures, and schedules across different sports. To determine if time to stabilization is a better predictor of future injury than clinical score, similar models will be constructed for the P&R clinical score, and we will assess each model's predictive ability using c-statistics (hypothesis 3b). Models with the highest c-statistic value will indicate the outcome most predictive of time to injury.

#### Sample Size Justification

We will have 80% power to detect a minimum hazard ratio of 1.53 per 1-s increase in stabilization time with a total sample size of 200 healthy participants, an event rate of 22%, and a standard deviation of 1 s using a Cox regression model at the 0.05 significance level. The event rate was estimated from the average number of athletes who experienced musculoskeletal injuries at the University of Utah from 2015 to 2018.

## Discussion

While a number of studies have examined static balance and gait after mTBI, reactive postural responses are an essential component of balance control for athletic performance and remain largely unstudied. An inability to accurately respond to postural disturbances could contribute to the increased musculoskeletal injury risk after mTBI. Therefore, the primary goal of this study is to determine if impaired reactive postural responses contribute to the increased musculoskeletal injury risk after mTBI. Using a two-phase approach, we will determine to what extent, and for how long, reactive postural responses are impaired after mTBI (phase 1), and we will determine the association between reactive postural responses and prospective musculoskeletal injury risk (phase 2). This study is unique in that it objectively measures reactive postural responses, an understudied component of balance, after mTBI.

Reactive postural responses are common in athletic competition and essential for athlete safety, but this domain of balance control has not been studied extensively after mTBI. Time to stability after dynamic movement can discriminate between those with history of mTBI and healthy controls (Lynall et al., 2020), suggesting that stability may be an important post-mTBI measure. However, no study has examined reactive postural responses in athletes with mTBI. We suspect the lack of knowledge concerning reactive postural responses after mTBI stems from two sources. First, reactive postural responses have been historically considered reflexive (subcortical) (Jacobs and Horak, 2007; Jacobs, 2014). However, it is likely that reactive postural responses rely on interaction among spinal circuits, the brainstem, and the cortex (Jacobs and Horak, 2007). As the focus of mTBI has traditionally been on cortical damage, postural responses may have been considered too automatic/reflexive to be disrupted by mTBI. The second possible reason for a gap in knowledge is logistical; traditional assessments of postural responses require sophisticated moving platforms (Zettel et al., 2008a,b) or tether-release (MacKey and Robinovitch, 2005; Inness et al., 2015) setups. Given some of the patient burden hurdles ingrained within clinical mTBI research, it is perhaps unsurprising that few studies have examined postural responses using these types of equipment. In fact, in designing this protocol, we considered implementing similarly controlled postural response paradigms (e.g., tether-release, moving platform, etc.). However, the need for a clinically feasible testing paradigm was critical to minimize the burden on student-athlete participants. Therefore, we selected the P&R test, a clinical test traditionally quantified using visual scoring. The notable weakness of this task is the relative simplicity for highly athletic individuals—few athletes should take multiple steps or struggle to recover. The use of wearable sensors is therefore essential and enables quantitative metrics of response latency, step length, and time to stabilization. The combination of wearable inertial sensors with the clinical P&R test facilitates assessments of reactive postural responses within an athletic training room with minimal added burden on the athlete. If we find athletes with recent mTBI take longer to regain stability or exhibit longer step latencies, it will provide evidence that mTBI impairs the reactive restoration of balance, demonstrating that mTBI affects all domains of balance control. Additionally, if we find differences between groups across time, it will support our hypothesis that postural responses are persistently impaired after mTBI. Such findings, coupled with the clinical feasibility of the P&R assessment, may spur the integration of postural response testing into the clinical management for mTBI. In the future, more work will be needed to develop outcome measures with established cutoff values for return-to-to play that could be incorporated into a testing battery.

The ability to carry out multiple tasks simultaneously, or to divide attention, is important in maintaining postural stability. Dual-task paradigms challenge the capacity for divided attention by requiring the participant to deal with competing cognitive and motor demands (Yogev-Seligmann et al., 2008). Because attentional capacity is limited, simultaneous performance of the tasks will cause a decline in one or both of the tasks (Posner and Boies, 1971; Kahneman, 1973). Correspondingly, dual-task deficits after mTBI during standing balance (Cavanaugh et al., 2005) and gait (Howell et al., 2013a, 2017) persist longer than motor tasks alone. If we find that reactive postural responses are impaired only in athletes with recent mTBI when performing a simultaneous cognitive task, it suggests that clinical evaluations of reactive balance control after mTBI should incorporate complex cognitive environments similar to gameplay. This finding would add support to existing literature demonstrating the need for dual-task assessments involving both physical and cognitive demands similar to those experienced during athletic competition.

A primary challenge in directly associating musculoskeletal injuries to underlying concussion deficits is adequate statistical power. To address this challenge, we considered the following to be true: if postural responses are associated with musculoskeletal within a population, the association will hold for a subgroup of that population. Therefore, if we find an association between reactive postural responses and the time to musculoskeletal injury in the general athletic population, irrespective of mTBI (phase 2), we expect that any impairment in reactive postural responses after mTBI will also associate with a faster time to injury. This assumption allows us to obtain adequate power for a longitudinal survival model within a reasonable timeframe (3 years). However, it does prompt a question about the validity of this assumption. The interpolation of a population-level association (all athletes) to a subgroup-level association (those with acute mTBI) will be directly tested by comparing the time to injury in athletes from phase 1 to test the validity of our assumption. Additionally, our concurrent two-phase approach will allow us to explore the association between changes in reactive postural responses from baseline-to-post-mTBI with time to injury using the small subset of athletes in which we will have baseline (pre-mTBI) and longitudinal post-mTBI measurements.

This study seeks to further our understanding of balance control after mTBI by examining a heretofore understudied, yet critically important aspect of balance, reactive postural responses. Through the innovative use of wearable sensor derived measures gathered during a clinical testing battery, the results of this study will improve our understanding of balance control post-mTBI and contribute to the foundation of relevant post-mTBI rehabilitation targets.

## Ethics Statement

Written informed consent was obtained from the individual(s) for the publication of any potentially identifiable images or data included in this article.

## Author Contributions

PF and LD led acquisition of funding from the PAC 12 that supports this project. AM and BC prepared the draft of the protocol with contributions from PF. AM, BC, RP, NF, AP, DC, NM, LD, and PF contributed to subsequent drafts and approved the final manuscript. All authors contributed to the article and approved the submitted version.

## Conflict of Interest

The authors declare that the research was conducted in the absence of any commercial or financial relationships that could be construed as a potential conflict of interest.
